# Dietary Nucleotides Supplementation and Liver Injury in Alcohol-Treated Rats: A Metabolomics Investigation

**DOI:** 10.3390/molecules21040435

**Published:** 2016-03-31

**Authors:** Xiaxia Cai, Lei Bao, Nan Wang, Meihong Xu, Ruixue Mao, Yong Li

**Affiliations:** 1 Department of Nutrition and Food Hygiene, School of Public Health, Peking University, Beijing 100191, China; shuiruoran8886@126.com (X.C.); kingsouth818@163.com (N.W.); xumeihong@bjmu.edu.cn (M.X.); rx334@163.com (R.M.); 2 Department of Clinical Nutrition, Peking University International Hospital, Beijing 102206, China; baolei6230@163.com

**Keywords:** alcohol, liver injury, metabolomics, nucleotides, oxidative stress

## Abstract

Background: Previous studies suggested that nucleotides were beneficial for liver function, lipid metabolism and so on. The present study aimed to investigate the metabolic response of dietary nucleotides supplementation in alcohol-induced liver injury rats. Methods: Five groups of male Wistar rats were used: normal control group (basal diet, equivalent distilled water), alcohol control group (basal diet, 50% alcohol (*v*/*v*)), dextrose control group (basal diet, isocaloric amount of dextrose), and 0.04% and 0.16% nucleotides groups (basal diet supplemented with 0.4 g and 1.6 g nucleotides kg^−1^ respectively, 50% alcohol (*v*/*v*)). The liver injury was measured through traditional liver enzymes, expression of oxidative stress markers and histopathological examination. Ultra-performance liquid chromatography quadrupole-time-flight mass spectrometry (UPLC-Q-TOF-MS) was applied to identify liver metabolite profiles. Results: Nucleotides supplementation prevented the progression of hepatocyte steatosis. The levels of total proteins, globulin, alanine aminotransferase, aspartate aminotransferase, total cholesterol triglyceride, as well as the oxidative stress markers altered by alcohol, were improved by nucleotides supplementation. Elevated levels of liver bile acids (glycocholic acid, chenodeoxyglycocholic acid, and taurodeoxycholic acid), as well as lipids (stearic acid, palmitic acid, oleic acid, phosphatidylcholine, and lysophosphatidylethanolamine) in alcohol-treated rats were reversed by nucleotides supplementation. In addition, supplementation with nucleotides could increase the levels of amino acids, including valyl-Leucine, l-leucine, alanyl-leucine and l-phenylalanine. Conclusion: These data indicate potential biomarkers and confirm the benefit of dietary nucleotides on alcoholic liver injury.

## 1. Introduction

Harmful use of alcohol is the leading cause of death, disease and injury globally with about 3.3 million alcohol-attributable deaths every year (5.9% of all deaths) [1]. Over half of liver diseases (mainly liver cirrhosis) are attributable to alcohol worldwide. Furthermore, 73.0% (males) and 59.8% (females) of liver cirrhosis cases are attributable to alcohol in China [1]. Although there have been many drugs to prevent or reverse the alcohol-induced liver injury in humans, they could produce some side effects, such as hepatotoxicity, nausea, abdominal pain, anorexia and diarrhea [2]. It has been reported that alcohol-induced liver injury is associated with metabolic alterations in liver, serum/plasma and urine [3,4]. Thus, seeking effective methods for regulating the metabolism may be of great clinical importance in both prevention and treatment of alcohol-induced liver injury.

Nucleotides (NTs) are basic units of nucleic acids and play important roles in most biological processes. They are the compositions of several coenzymes and play key roles in energy metabolism and biosynthetic pathways [5]. They also serve as conditionally essential nutrients under certain circumstances such as clinical situations, a period of insufficient nutrient intake, a rapid growing phase, and during development [6]. Beneficial effects of NTs on lipid metabolism [5], liver damage [7], immune function [8], and intestinal function and growth [9,10] have been explored and discussed widely, whereas the effects of NTs on alcohol-induced liver injury are still unknown. Therefore, further evidence of health effects of NTs supplementation and exploration of the possible mechanisms are needed.

Metabolomics analysis is a holistic qualitative and quantitative assessment of low-molecular-weight metabolites in biological samples. In mammals, metabolomics is applied to the study of disease, drugs and nutrients administration, normal physiological processes and so on. It can provide valuable information as how to define pathophysiological processes and discover biomarkers [11,12]. Up to now, endogenous metabolic changes caused by NTs supplementation are reported poorly. Only two studies investigated the fatty acid alterations of plasma and liver microsomes in thioacetamide- or carbon tetrachloride- treated rats fed with nucleotides. However, both of them used only gas-liquid chromatography [13,14]. Dietary nucleotides were confirmed to have the capability to correct fatty acid alterations in rats with liver damage in those studies. No other related studies are reported.

Gas (GC) or liquid (LC) chromatography (particularly ultra-performance LC, UPLC) coupled with mass spectrometry (MS) or nuclear magnetic resonance (NMR) spectroscopy is still the dominant analytical platforms for metabolomics studies [11,12]. Among the various methods, LC-MS has been welcomed because of its high throughput, soft ionization, and good metabolites coverage [15]. Furthermore, UPLC-MS is quite suitable for the detection and identification of small-molecular-weight compounds [12,16].

Therefore, the present study aimed to use the ultra-performance liquid chromatography quadrupole-time-flight mass spectrometry (UPLC-Q-TOF-MS) approach to investigate the metabolic alterations of dietary nucleotides supplementation in rats treated by alcohol, and multivariate statistical analysis was performed for detecting the novel biomarkers of dietary nucleotides supplementation. The results might be considered as new information on the identification of biomarkers and review the benefits of dietary nucleotides on liver injury.

## 2. Results

### 2.1. Biochemical Indices in the Serum

As shown in Table 1, serum total proteins (TP) and globulin (GLB) levels were significantly decreased in the alcohol control group, compared with the dextrose control group (*p* < 0.01). While serum alanine aminotransferase (ALT), aspartate aminotransferase (AST), total cholesterol (TC), triglyceride (TG) levels and albumin:globulin (A:G) ratio of the alcohol control group was higher than that of dextrose control group (*p* < 0.05 or *p* < 0.01). After NTs treatment (especially 0.16% NTs), the parameters above were significantly reversed (*p* < 0.05 or *p* < 0.01). The serum total bilirubin (TBIL) level of the alcohol control group was higher than that of dextrose control group, but there was no significance. 0.16% NTs decreased the TBIL level significantly (*p* < 0.05 compared with alcohol control group).

### 2.2. Histopathological Result

**Figure 1:**
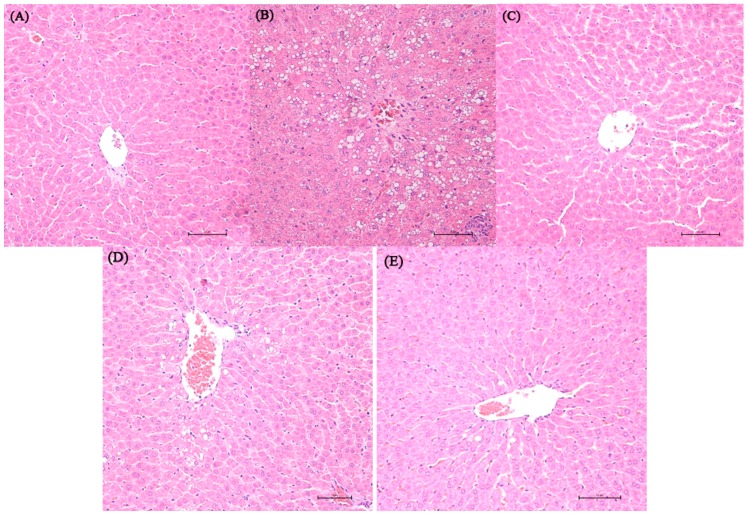
Effect of nucleotides (NTs) on liver histology in rats. Representative photomicrographs with H & E staining reveal histopathological changes of liver from normal control rats, dextrose control rats and alcohol control rats with or without nucleotides treatment (400×). (**A**) Normal control group; (**B**) Alcohol control group; (**C**) Dextrose control group; (**D**) 0.04% NTs group; (**E**) 0.16% NTs group.

Representative photomicrographs exhibiting liver pathology (H & E staining) are presented in Figure 1. Compared with normal control and dextrose control rats, alcohol exposure caused disappearance of hepatic cord, irregular arrangement of the hepatocytes and hepatocyte steatosis. However, no inflammation and apoptosis were observed in the alcohol control group. NTs feeding in alcohol-treated rats remarkably reduced the number of steatotic hepatocytes. More regular hepatic cords and hepatocytes with clear border were shown in the liver of 0.04% NTs- and 0.16% NTs-treated rats.

### 2.3. Oxidative Stress in Liver Tissue

**Figure 2:**
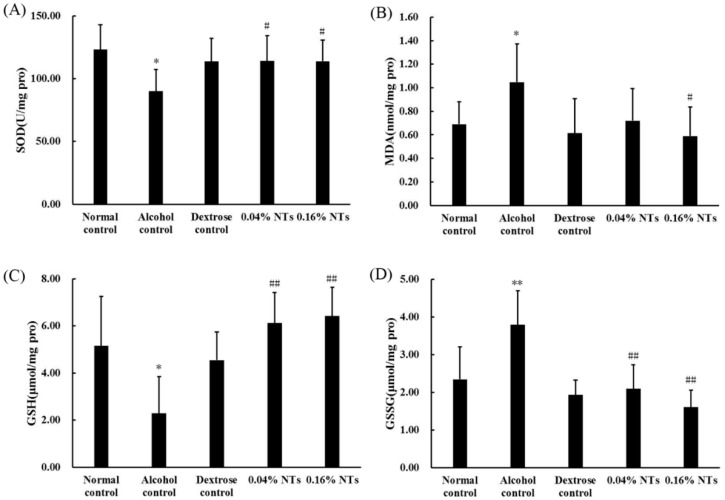
Effects of nucleotides (NTs) on oxidative stress in liver tissues of rats. (**A**) Superoxide dismutase (SOD) activity; (**B**) malondialdehyde (MDA); (**C**) reduced glutathione (GSH); and (**D**) oxidized glutathione (GSSG) levels in liver tissues of rats. Values were expressed as the mean ± standard deviation of ten rats per group. Mean values were significantly different from those of the dextrose control group: * *p* < 0.05; ** *p* < 0.01. Mean values were significantly different from those of the alcohol control group: ^#^ *p* < 0.05; ^##^ *p* < 0.01.

As shown in Figure 2, the superoxide dismutase (SOD) activity and reduced glutathione (GSH) levels significantly decreased in liver tissues of alcohol control rats compared with those of dextrose control rats (*p* < 0.05). The malondialdehyde (MDA) and oxidized glutathione (GSSG) contents in the liver tissues of alcohol control rats were significantly higher than those of dextrose control rats (*p* < 0.05 for MDA, *p* < 0.01 for GSSG). However, the intervention of NTs exhibited protection against alcohol-induced SOD and GSH depletion (both NTs groups: *p* < 0.05 for SOD, *p* < 0.01 for GSH). MDA and GSSG levels were also lowered by the NTs intervention significantly (MDA: *p* < 0.05 in the 0.16% NTs group, compared with the alcohol control group; GSSG: *p* < 0.01 in both NTs groups, compared with the alcohol control group).

### 2.4. Metabolite Detection

Metabolites in liver samples from dextrose control, alcohol control, 0.04% NTs and 0.16% NTs rats were profiled by UPLC/Q-TOF MS. The typical base peak intensity chromatograms are shown in supplementary figure (Figure S1). We filtered metabolites by quality control samples and deleted the coefficient of variation values of the metabolites which were less than 30%. 588 metabolites (positive ion mode) and 209 metabolites (negative ion mode) were reserved.

### 2.5. Multivariate Data Analysis

**Figure 3:**
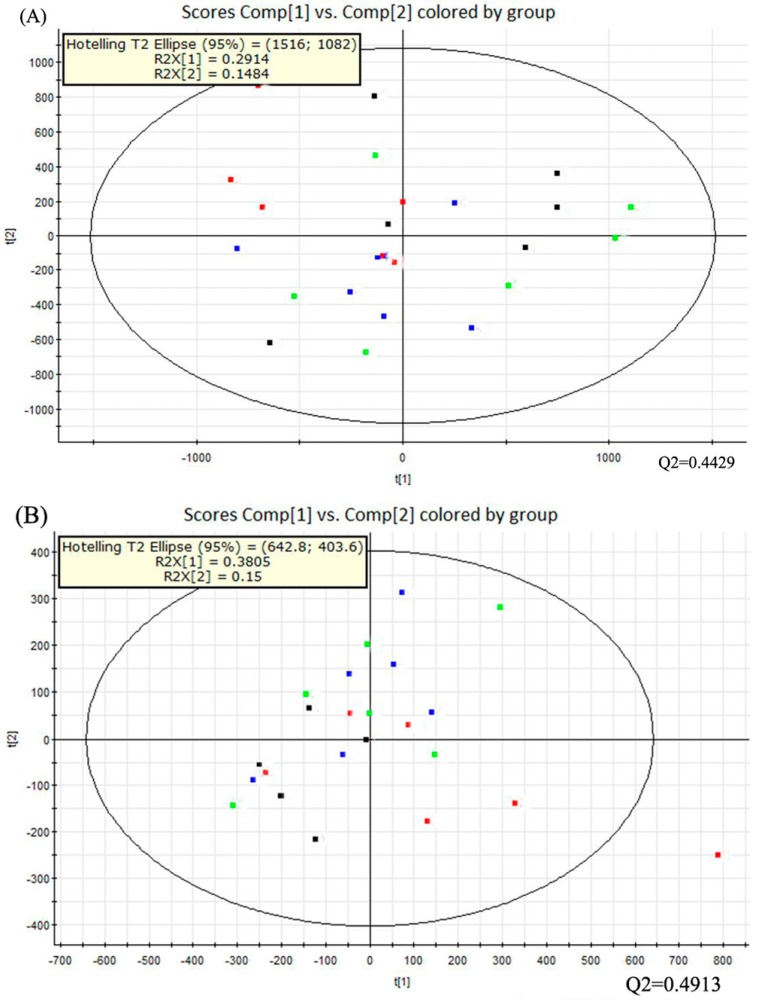
PCA score plots of the four tested groups from UPLC-Q-TOF-MS profiling data. The PCA score plot showed that different liver samples were distributed into different areas; all samples were in the Hostelling T2 ellipse. (**A**) PCA score plot from positive ion mode; (**B**) PCA score plot from negative ion mode. PCA, Principal component analysis; UPLC-Q-TOF-MS, Ultra-performance liquid chromatography- quadrupole-time-of-flight-mass spectrometry. (■: Dextrose control; ■: Alcohol control; ■: 0.04%NTs; ■: 0.16%NTs).

Multivariate data analysis was performed with the principal component analysis (PCA) method first (Figure 3). It showed the distribution of the original data. The separation of dextrose control, alcohol control, 0.04% NTs and 0.16% NTs groups were not obvious in the PCA score plots in positive ion mode; three samples of alcohol control group were away from other samples in negative ion mode. The PCA score plots accounted for 29.14% (t[1]) and 14.84% (t[2]) of the variations with ESI+, 38.05% (t[1]) and 15% (t[2]) of the variations with negative ion mode. The R2X and Q2 values were 0.4398 and 0.4429 with positive ion mode respectively; The R2X and Q2 values were 0.5306 and 0.4913 with negative ion mode respectively.

**Figure 4:**
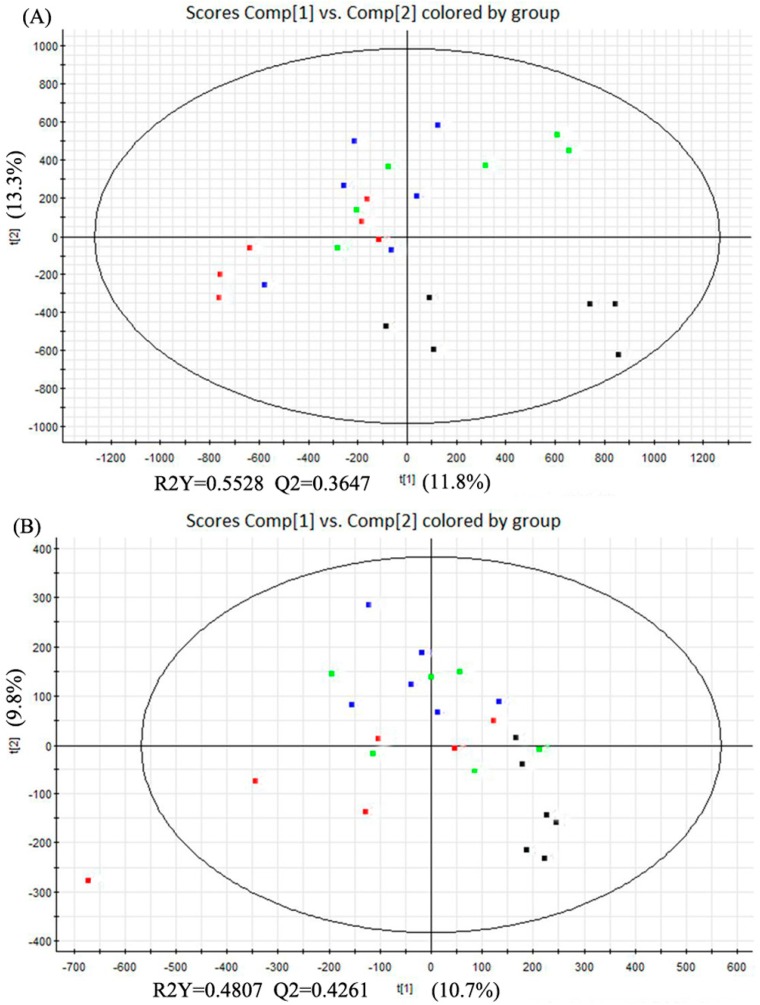
PLS-DA score plots of the four tested groups from UPLC-Q-TOF-MS profiling data. (**A**) PLS-DA score plot from positive ion mode; (**B**) PLS-DA score plot from negative ion mode. PLS-DA, Partial least squares discriminant analysis; UPLC-Q-TOF-MS, Ultra-performance liquid chromatography- quadrupole-time-of-flight-mass spectrometry. (■: Dextrose control; ■: Alcohol control; ■: 0.04%NTs; ■: 0.16%NTs).

The partial least squares discriminant analysis (PLS-DA) method was used to further study the variance between different groups. As shown in Figure 4, PLS-DA score plots from the first two dimensions explained 11.8% (t[1])and 13.3% (t[2]) of the variations with positive ion mode, and 10.7% (t[1]) and 9.8% (t[2]) of the variations with negative ion mode. In positive ion mode, PLS-DA score plots of the liver tissue showed distinct clustering trend between dextrose control group and another three groups, but separation of the remaining three groups were not obvious—only three samples of alcohol control group were away from 0.04% and 0.16% NTs groups. 0.04% NTs and 0.16% NTs groups were much closer to dextrose control group than alcohol control group. In negative ion mode, samples of alcohol control group were slightly separated from the remaining three groups, although three alcohol control samples meshed with the rest of the groups. Among the metabolites, 58 metabolites with positive ion mode and 16 metabolites with negative ion mode were selected by *p-*value (<0.05) in the liver samples.

### 2.6. Analysis of Potential Biomarkers

**Figure 5:**
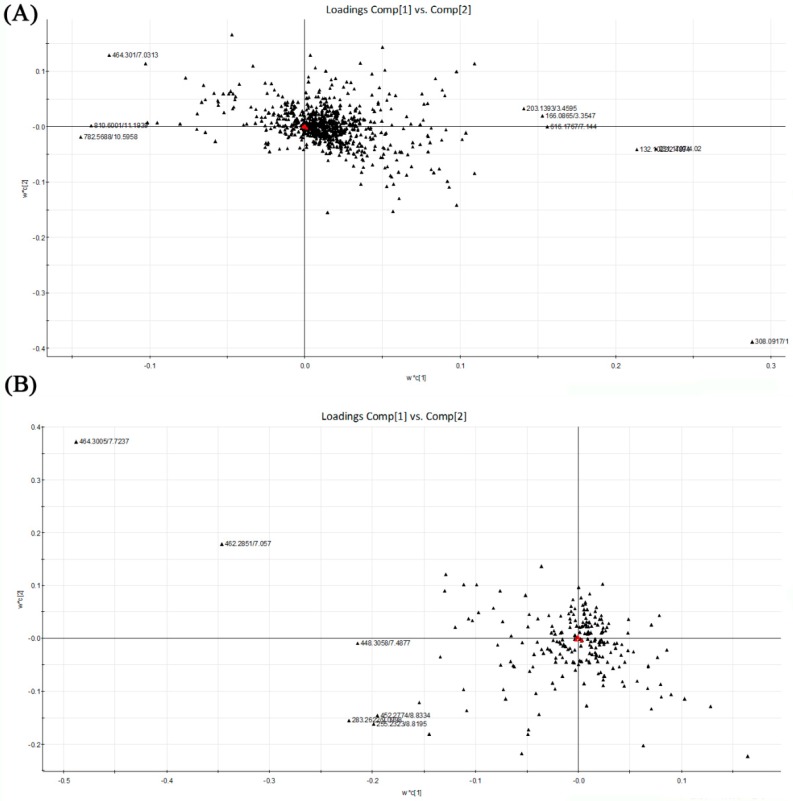
PLS-DA loading plots of the four tested groups from UPLC-Q-TOF-MS profiling data. Red triangles represent responses; Blacks represent X variables. (**A**) PLS-DA loading plot from positive ion mode; (**B**) PLS-DA loading plot from negative ion mode. PLS-DA, Partial least squares discriminant analysis; UPLC-Q-TOF-MS, Ultra-performance liquid chromatography-quadrupole-time-of-flight-mass spectrometry.

Based on the PLS-DA, the loading plots were constructed (Figure 5). It showed important variables which made significant contributions to the differences among the four groups. The significant variables were situated far from the origin. Although there were not obvious separations among the tested groups, the loading plots also showed that the metabolites located on the left were more abundant in alcohol control group than in dextrose control group. Accompanied by the loading plots, variable importance in the partial least squares project (VIP) value was also applied for the identification of potential biomarkers. Variables with VIP value larger than 2.00 represented higher influence on the classification. These variables were selected as potential biomarkers last. The *p*-value from one-way analysis of variance (ANOVA) was used to find the potential biomarkers that contributed to the discrimination. *p* < 0.05 was considered to be significant. Table 2 shows the top 15 metabolites with VIP value >2.00 and *p* < 0.05 in liver samples respectively. It indicated that these metabolites were highly relevant to differences among sample groups.

The peak area intensities of these metabolites are shown in Table 2. Of these metabolites, the levels of three bile acids (glycocholic acid, chenodeoxyglycocholic acid and taurodeoxycholic acid), three fatty acids (stearic acid, palmitic acid and oleic acid), phosphatidylcholine [PC (36:4)] and two lysophosphatidylethanolamine (LysoPE (16:0) and LysoPE (18:0)) were significantly increased in alcohol control group compared with dextrose control group. Whereas the levels of three amino acids (valyl-leucine, l-leucine, alanyl-leucine) were significantly decreased in the alcohol control group compared with dextrose control group. However, compared with alcohol control group, the levels of chenodeoxyglycocholic acid, stearic acid, palmitic acid, oleic acid, PC (36:4), LysoPE (16:0) and LysoPE (18:0) were significantly decreased in both NTs groups. Moreover, the level of taurodeoxycholic acid was significantly decreased and those of valyl-Leucine, l-leucine, alanyl-leucine and l-phenylalanine were significantly increased only in 0.04% NTs group. In addition, the level of glutathione was lower in alcohol control group than that in dextrose control group.

## 3. Discussion

To the best of our knowledge, the present study was the first to report that dietary NTs supplementation improved the liver metabolite profile and then relieved liver injury in alcohol-treated rats. Numerous molecules, referring to bile acid metabolism, lipid metabolism and amino acid metabolism, were changed in the alcohol control group and then partially normalized by supplementation of NTs.

There were different methods to build an alcoholic liver injury model. In a study by Mutle *et al.* [17], SD rats were given 50%–60% (*v*/*v*) alcohol intragastrically at an initial dose of 2 g/kg body weight per day, the dose was gradually increased within two weeks to a maintenance dose of 8 g/kg body weight per day for another eight more weeks. Rats were induced to alcoholic steatohepatitis. Here we developed the liver injury model with a progressive increasing dose (2–8 g/kg body weight) for two weeks and a maintenance dose of 8 g/kg body weight for another four weeks. The liver of rats showed hepatocyte steatosis after alcohol treatment. Afterwards, NTs supplementation could alleviate hepatic steatosis. The intake of NTs could alleviate the accumulation of lipid droplets in thioacetamide-induced liver injury [7]. The possible reason was that NTs supplementation could increase the intracellular concentrations of metabolic nucleotides, such as CDP-choline [18]; CDP-choline is a nucleotide necessary for phospholipid synthesis and lipoprotein assembly [18,19]. Elevated serum ALT and AST have been considered as the indicators of alcoholic liver injury [20]. The present results showed increased ALT and AST levels following alcohol treatment, which proved the liver injury of alcohol-treated rats. The NTs showed protective effect on alcohol-induced liver injury by reversing these above markers.

Acute and chronic ethanol treatments induce the production of reactive oxygen species (ROS), deplete cellular antioxidant capacity, and evoke oxidative stress in many tissues, especially in the liver [21]. Abnormal oxidative stress biomarkers, such as MDA, SOD and GSH, are observed in alcohol-treated animals. MDA is the most abundant product of lipid peroxidation. A previous study in our laboratory indicated that MDA level was increased significantly in rats exposed to alcohol [22]. SOD is one of the important ROS scavengers, and catalyzes the process of superoxide anion radical (O_2_^−^) conversion to hydrogen peroxide (H_2_O_2_). Its activity was inhibited after alcohol administration [23]. GSH is another ROS detoxicant in the liver. Previous studies demonstrated that hepatic glutathione level was depleted in alcohol-treated rats [24,25]. In the present study, alcohol-treated rats showed higher MDA and GSSG levels, lower GSH level and SOD activity in the liver than dextrose control rats, whereas NTs supplementation could reverse the above oxidative stress biomarkers. There have been no other reports confirming the effects of dietary NTs on alcohol-induced liver injury, but a similar antioxidative activity of dietary NTs on aged rats was confirmed in our laboratory [26]. Moreover, the intake of NTs could restore mitochondrial function and enhance the liver redox state in thioacetamide-induced liver injury [18]. Based on the results above, it can be concluded that NTs have the capacity to restore alcohol-induced liver injury partly due to the inhibition of oxidative stress.

Liver is one of the main organs for bile acid synthesis, which plays a key role in multiple biochemical pathways, such as lipid, cholesterol and glucose metabolism, vitamins absorption and so on. However, elevated levels of hepatic bile acids are representations of alcoholic liver disease pathogenesis and also promote liver injury [27,28]. Aranha *et al.* [29] showed that alcoholic steatohepatitis had higher total bile acids, chenodeoxycholic acid and deoxycholic acid than controls. Moreover, classical pathway of bile acids synthesis was up-regulated in alcohol-treated human hepatocytes [30]. In the present study, striking increases in the primary bile acids including glycocholic acid and chenodeoxyglycocholic acid and the secondary bile acid with taurodeoxycholic acid were observed in alcohol control group. In addition, the present study demonstrated that higher serum cholesterol was also observed in alcohol control group. Therefore, the increased levels of bile acids may be due to the accumulation of lipid and cholesterol in liver and the excretory dysfunction of liver in alcohol-treated rats. In addition, increased bile acids levels could accelerate liver injury, leading to a vicious cycle. NTs supplementation could inhibit the rise of liver bile acids, indicating its ability of lowering bile acids.

In the present study, fatty acid and phospholipids biosynthesis and metabolism were also disturbed by alcohol treatment according to the metabolomics analysis. Notably, NTs supplementation significantly decreased liver saturated fatty acids (SFA) including stearic acid and palmitic acid. Besides, oleic acid, a monounsaturated fatty acid were also decreased significantly in NTs group compared with alcohol control group. Excess alcohol intake inhibits fatty acid oxidation [31,32]. Hepatic steatosis characterized by increased SFAs promoted liver injury in animal model of partial hepatectomy [33]. Hernández *et al.* [34] suggested that free fatty acids (oleic and palmitic acid) enhanced the oxidative damage in ethanol-treated VL-17A cells. Fontana *et al.* [13] suggested that an NT-enriched diet could correct the increased levels of palmitic, oleic, linoleic and arachidonic acids induced by thioacetamide, which was consistent with our study. However, more evidences are needed to confirm whether stearic acid, palmitic acid and oleic acid could be biomarker of alcoholic liver injury.

Glycerophospholipid metabolites, including PC and LysoPE are key components of the lipid bilayer of cells, as well as being involved in metabolism and signaling. It has been reported that various PCs and LysoPEs were significantly increased in alcohol-treated mice or rats [35,36,37]. Excessive alcohol intake also decreases the activity of lecithin cholesterol acyltransferase (LCAT), which is secreted from the liver, leading to serum PCs increase [38]. LCAT can catalyze the transfer of fatty acids of position sn-2 of phosphatidylcholine to the free cholesterol in plasma, with formation of cholesterol esters and lysophosphatidylcholine. A previous study suggested that NTs increased high-density lipoprotein cholesterol serum level and decreased low-density lipoprotein cholesterol serum level in preterm neonates [39]. Moreover, NTs also increased the plasma LCAT activity in preterm infants [40]. Notably, our data showed that the elevated levels of PC (36:4), LysoPE (16:0) and LysoPE (18:0) observed in the liver of alcohol-treated rats were remarkably decreased by NTs supplementation. The elevated TG and TC serum levels were also reversed by NTs supplementation. All of the above implied that lipid metabolism was partly restored by NTs supplementation.

Another important finding from the present study was that NTs supplementation affected metabolism and biosynthesis of amino acid in alcohol-treated rats. Results demonstrated that amino acid metabolism were disturbed by alcohol, leading to lower the levels of l-leucine, valyl-leucine, alanyl-leucine and l-phenylalanine in the liver, and the decreases were reserved by NTs supplementation. Of the four amino acids significantly decreased in alcohol control group, leucine, one of branched-chain amino acids (BCAA), is essential nutrients only from food [41]. BCAAs are recommended for the nutritional therapy of alcoholic liver disease and complications [42,43]. They have also been proved to reduce oxidative stress [44]. Decreased liver concentration of leucine in alcohol-treated rats in the present study is consistent with another finding [45]. Phenylalanine, one of the aromatic amino acids, was also decreased significantly in alcohol control group compared with in dextrose control group. However, some studies showed that patients with alcoholic cirrhosis or alcoholic liver disease had significantly increased serum levels of phenylalanine compared with controls [45,46]. Another study did not observe statistically different aromatic amino acids between alcoholic patients and controls [47]. Further studies are needed to confirm the alteration of aromatic amino acids after alcohol treatment. Bode *et al.* [48] suggested that exposure of the small intestine to alcohol can impair the absorption of some nutrients including amino acid. This may be one reason for the decreased amino acids in alcohol liver injury. Many studies suggested that NTs supplementation could augment the synthesis of protein, increase the concentration of total protein and albumin [13,18,19]. Since *de novo* synthesis of NT is an energy-intensive process, earlier study suggested that exogenous NTs may save energy and intermediary metabolites via salvage synthesis. It may be good for the turnover of and synthesis of proteins, the differentiation and proliferation of cells and the recovery of the intestine [49,50]. Our data showed that NTs supplementation significantly increased the concentration of leucine and phenylalanine in the liver, indicating other potential biomarkers for the effects of NTs on alcoholic liver injury.

## 4. Materials and Methods

### 4.1. Materials and Reagents

Basal diet (AIN-93G rodent diet) and the NTs-supplemented diet (basal diet supplemented with 0.4 g and 1.6 g NTs·kg^−1^ respectively) were produced by HFK Bioscience Co. Ltd. (Beijing, China). NTs were provided by Zhen-Ao Biotechnology Ltd. Co. (Dalian, China). The NTs content was more than 99%. The NTs which were derived from brew yeast RNA, included 22.8% 5′-adenosine monophosphate (5′-AMP), 26.6% 5′-cytidine monophosphate (5′-CMP), 20.4% 5′-guanosine monophosphate (5′-GMP) Na_2_ and 30.2% 5′-uridine monophosphate (5′-UMP)Na_2_. Ethanol was of analytical reagent grade (Beijing Chemical Company, Beijing, China).

### 4.2. Animals

The Peking University Animal Research Committee (www.lab.pku.edu.cn) approved the protocols before starting. Fifty male Wistar rats (300–350 g) were purchased from the Animal Service of Health Science Center, Peking University. Rats were housed 2 per cage in a filter-protected, air-conditioned room with constant temperature (21–25 °C), relative air humidity (40%–50%) and a 12 h light/dark cycle (lights on at 07:30–19:30 h). All animal treatment and experimental procedures were in accordance with the Principle of Laboratory Animal Care (NIH publication No. 85–23, revised 1985).

### 4.3. Experimental Design

Rats were acclimatized to new environment for 2 weeks, and then were randomly divided into 5 groups. Group 1 (*n* = 10, normal control), Group 2 (*n* = 10, Alcohol control) and Group 3 (*n* = 10, Dextrose control) were fed the basal diet. Group 4 (*n* = 10, 0.04% NTs) and Group 5 (*n* = 10, 0.16% NTs) were fed with basal diet supplemented with 0.4 g and 1.6 g NTs·kg^−1^ respectively. The rats in Group 2, 4, and 5 were given 50% alcohol (*v*/*v*) intragastrically (2–3 mL) twice a day. The dose was started with 2 g/kg per day and gradually increased to 8 g/kg per day within 2 weeks. This dose was maintained for up to 4 weeks. Group 1 and Group 3 were orally administered with equivalent distilled water and isocaloric dextrose for control respectively. During the experimental period, all groups were allowed free access to water and food. At last, after alcohol treatment for 12 h, animals were anesthetized by diethyl ether. Blood was obtained from femoral artery; serum was separated (3000 g for 20 min at 4 °C) for biochemical assays. The liver were also obtained for biochemical assay and metabolomics study.

### 4.4. Biochemical Assay

The levels of ALT, AST, TP, ALB, GLB, TBIL, TC, and TG in serum were detected by Olympus AU400 automatic biochemistry analyzer (Olympus, Tokyo, Japan). The SOD activity, GSH, GSSG and MDA levels in liver tissues were determined with SOD, MDA and GSH/GSSG detection kits according to the manufacturer’s protocols. All detection kits were purchased from Beyotime Institute of Biotechnology (Beijing, China).

### 4.5. Histopathological Observation

Liver tissues for histopathological observation were acquired from the same lobe. 10% (*v*/*v*) formaldehyde-fixed, paraffin-embedded liver sections (5 mm-thick) were stained with haematoxylin and eosin and then were studied with an Olympus IX70 inverted microscope (Olympus, Tokyo, Japan).

### 4.6. Sample Preparation

100 mg liver tissue was homogenized with 1 mL distill water/acetonitrile (1:1) three times by a Qiagen Tissuelyser at 4 °C. Samples were extracted for 30 min. The mixture was centrifuged at 12,000 rpm for 5 min at 4 °C. The supernatant (600 μL) was taken up and was dried down in a centrifugal vacuum evaporator for 4 h. 100 μL distill water/acetonitrile (1:1) was added to each dry sample, follow by vigorous vortex for 40 s. Each sample was centrifuged at 12,000 rpm for 5 min at 4 °C. 80 μL supernatant was transfer to 200 μL inner-lining tube for future use.

### 4.7. Ultra-Performance Liquid Chromatography

Ultra-performance liquid chromatography was performed using an Acquity system coupled with a Q-TOF premier (Waters Corporation, Milford, MA, USA). The chromatographic separation was performed on a Waters ACQUITY UPLC High-strength silica (HSS) T_3_ column (1.7 μm, 2.1 mm × 100 mm) operating at 40 °C. The flow rate was at 0.3 mL/min of a binary mobile phase system consisting of water containing 0.1% (*v*/*v*) formic acid (A) and acetonitrile (B). The gradient elutions were described in supplementary file and Table (Table S1). The injection volume was 3 μL.

### 4.8. Mass Spectrometry

MS was performed on a Waters Xevo G2 Q-TOF mass spectrometer (Waters Corporation) coupled with an electrospray ionization (ESI) and quadrupole time-of-flight analyzer. Ionization was performed in both positive and negative modes ionization. The data were collected for each sample from a mass range of 50 *m*/*z* to 1200 *m*/*z*. High purity nitrogen (N_2_) was used in gas circuit. Source parameters were as follows: capillary voltage, 3000 V; sampling cone voltage, 25 V; desolvation temperature, 350 °C; desolvation gas flow, 800 L/h; cone gas flow, 50 L/h; source temperature, 100 °C. To ensure experimental accuracy and reproducibility, saline blank samples and quality control samples were used. Briefly, one quality control sample was injected at regular intervals (every six samples). Coefficient of variation of metabolite feature intensities (peak area) among quality control samples should be lower than 30%. This ensured accurate mass measurements.

### 4.9. Data Processing

Markerlynx 4.1 software (Waters Corporation) was used for raw peaks exacting, data baselines filtering, baseline calibration, peak alignment, peak identification and integration of peak area. Each chromatographic peak was identified by retention time (RT) and mass to charge ration (*m*/*z*) data pairs. Following this, the signal intensity was normalized by Pareto scaling. The resulting data array comprising the variables plasma or liver sample, RT, *m*/*z* values, and peak area intensity, were used for further multivariate statistical analysis. It includes PCA and PLS-DA. PCA and PLS-DA are the unsupervised and supervised multivariate statistical methods, respectively. They were performed with the SIMCA-P^+^ software package (Umetrics EZ info 2.0, Umea, Sweden) in the Markerlynx software. R2X (cum), R2Y (cum) and Q2 (cum) were used to evaluate the quality of PCA and PLS-DA model. R2 shows the goodness of fit. Q2 indicates the model predictive capability [51]. Loading plots and VIP values greater than 2.0 were also used to select potential biomarkers. One-way analysis of variance (ANOVA) was used to find the differential metabolites that contributed to the discrimination. *p* < 0.05 was considered to be significant.

### 4.10. Identification of Biomarkers

Commercial databases, including Human Metabolome and Database (HMDB) (http://www.hmdb.ca/), METLIN metabolite database [52] and Kyoto Encyclopedia of Genes and Genomes (KEGG) [53]() were utilized for biomarker identification according to their RT, *m*/*z* and fragment ions.

### 4.11. Statistical Analysis

Data about protein, lipid and bilirubin levels and chromatographic peak area intensity were analyzed by SPSS 13.0 for Windows (SPSS, Inc., Chicago, IL, USA). All of the above data were presented as means and standard deviations. Homogeneity of them was confirmed by SPSS software. If variances were equal, data were analyzed by means of one-way analysis of variance (ANOVA) with least significant difference (LSD) tests; Otherwise, Tamhane’s T2 test was used. *p* < 0.05 was considered to be significant.

## 5. Conclusions

This current research demonstrated that NTs supplementation could partially, but not completely, alleviate alcoholic liver injury and moderated metabolism. The results indicated that some potential biomarkers in regard to bile acid metabolism, lipid metabolism and amino acid metabolism were identified and suggested to potential benefits and mechanisms because of the alteration of these metabolites. Further studies are necessary to determine the optimal dose of nucleotides supplementation for preventing alcoholic liver injury.

## Figures and Tables

**Table 1:** Effects of dietary nucleotides (NTs) on aminotransferase, protein, lipid and bilirubin levels in the serum of rats (Mean values and standard deviations, n 10 per group).

Parameters	Normal Control		Alcohol Control		Dextrose Control		0.04% NTs		0.16% NTs	
	Mean	SD	Mean	SD	Mean	SD	Mean	SD	Mean	SD
ALT (U/L)	32.89	9.98	47.44 *	25.53	32.89	7.99	31.44 ^#^	9.54	33.00 ^#^	7.33
AST (U/L)	62.58	13.61	85.81 **	26.46	58.49	15.92	54.11 ^##^	14.46	52.91 ^##^	12.59
TP (g/L)	73.56	3.55	66.78 **	3.40	73.33	4.43	69.20	5.55	71.81 ^#^	6.19
ALB (g/L)	38.22	1.37	36.86	1.93	38.16	1.25	37.19	2.15	37.54	2.12
GLB (g/L)	35.44	2.55	29.92 **	1.63	35.18	3.54	32.01	3.84	34.27 ^##^	4.23
A:G ratio	1.08	0.07	1.23 **	0.04	1.09	0.09	1.17	0.10	1.11 ^##^	0.10
TBIL (μmol/L)	2.01	0.40	2.46	0.50	2.09	0.27	2.24	0.46	2.03 ^#^	0.42
TC (mmol/L)	2.07	0.38	2.80 *	0.61	2.22	0.34	2.34 ^#^	0.26	2.29 ^#^	0.32
TG (mmol/L)	1.30	0.29	1.89 *	0.73	1.32	0.28	1.66	0.55	1.31 ^#^	0.49

ALT, alanine aminotransferase; AST, aspartate aminotransferase; TP, total protein; ALB, albumin; GLB, globulin; A:G, albumin:globulin; TBIL, total bilirubin; TC, total cholesterol; TG, triglyceride. Mean values were significantly different from those of the dextrose control group: * *p* < 0.05; ** *p* < 0.01. Mean values were significantly different from those of the alcohol control group: ^#^ *p* < 0.05; ^##^ *p* < 0.01.

**Table 2:** Representative differential metabolites of liver that contributed to the separation among dextrose-, alcohol- and NTs-treated rats derived from UPLC-TOF-MS analysis (n 6 per group).

Compounds	RT(min)_*m*/*z*	VIP	Peak Area Intensity				Pathway
			Dextrose Control	Alcohol Control	0.04% NTs	0.16% NTs	
Glycocholic acid	7.7237_464.3005	5.59	8463.81 ± 7415.40	31,624.49 ± 15,933.73 **	26,484.27 ± 18,211.81	31,964.50 ± 15,863.17	Primary bile acid biosynthesis; Secondary bile acid biosynthesis; Bile secretion
Chenodeoxyglycocholic acid	7.4877_448.3058	2.20	143.49 ± 278.14	5104.29 ± 833.33 **	1725.13 ± 1099.87 ^##^	2587.23 ± 725.60 ^##^	Primary bile acid biosynthesis; Secondary bile acid biosynthesis; Bile secretion
Taurodeoxycholic acid	7.057_462.2852	3.57	498.07 ± 915.56	10,764.23 ± 2193.21 **	5839.72 ± 1832.43 ^##^	9480.95 ± 2332.42	Primary bile acid biosynthesis; Secondary bile acid biosynthesis; Bile secretion
Stearic acid	9.0998_283.2622	3.18	970.48 ± 1097.17	7191.91 ± 1879.98 **	2026.56 ± 1676.70 ^##^	1543.03 ± 758.30 ^##^	Fatty acid biosynthesis; Biosynthesis of unsaturated fatty acids
Palmitic acid	8.8195_255.2323	3.04	3510.95 ± 3186.85	9469.90 ± 3202.26 **	5350.94 ± 3418.26 ^#^	3445.33 ± 1681.00 ^##^	Fatty acid metabolism; Fatty acid elongation; Biosynthesis of unsaturated fatty acids
Oleic acid	8.9027_281.248	2.87	1951.77 ± 1228.59	5639.40 ± 2538.87 **	2738.06 ± 2399.18	1113.86 ± 1067.94	Fatty acid biosynthesis; Biosynthesis of unsaturated fatty acids
LysoPE(16:0)	8.8334_452.2774	2.87	575.73 ± 509.70	5241.31 ± 925.57 **	1375.93 ± 1208.68 ^##^	723.85 ± 558.81 ^##^	NUM
LysoPE(18:0)	9.0983_480.3084	2.31	95.30 ± 152.35	3245.92 ± 340.42 **	356.80 ± 402.64 ^##^	248.25 ± 262.90 ^##^	NUM
PC(36:4)	10.5958_782.5688	3.02	4685.98 ± 7215.85	19,441.81 ± 14,285.09 *	5760.80 ± 7153.05^#^	4855.63 ± 3996.21 ^#^	Glycerophospholipid metabolism; Linoleic acid metabolism; Arachidonic acid metabolism; alpha-Linolenic acid metabolism; Biosynthesis of secondary metabolites
Valyl-Leucine	4.0200_231.1707	4.23	82,347.30 ± 15,277.29	56,815.96 ± 11,255.82 *	82,653.81 ± 17,597.53 ^#^	55,640.86 ± 20,887.14	NUM
l-Leucine	2.4894_132.1022	3.98	77,270.19 ± 19,282.77	46,539.18 ± 11,543.08 *	72,540.58 ± 23,121.88 ^#^	63,797.58 ± 28,472.31	Valine, leucine and isoleucine biosynthesis and degradation; Biosynthesis of secondary metabolites; Biosynthesis of amino acids; Protein digestion and absorption; ABC transporters
Alanyl-Leucine	3.4595_203.1393	3.12	30,881.02 ± 5469.33	20,193.62 ± 6616.19 *	33,593.60 ± 11,567.90 ^#^	24,107.26 ± 10,181.59	NUM
l-Phenylalanine	3.3547_166.0865	3.20	62,390.84 ± 10,449.98	50,789.46 ± 5932.05	66,602.95 ± 17,891.97 ^#^	51,682.26 ± 10,729.99	Phenylalanine metabolism; Phenylalanine, tyrosine and tryptophan biosynthesis; Biosynthesis of secondary metabolites; Biosynthesis of amino acids; ABC transporters; Protein digestion and absorption
l-Phenylalanine (Fragment)	3.3594_120.081	3.81	52,558.23 ± 8271.70	45,012.33 ± 5773.60	61,986.71 ± 16,794.77 ^#^	51,397.55 ± 9687.96	
Glutathione	1.6432_308.0917	8.02	123,866.59 ± 72,656.44	53,551.25 ± 36,343.10 *	62,413.02 ± 38,243.30	78,404.52 ± 52,299.03	Cysteine and methionine metabolism; Glutathione metabolism; Metabolic pathways; ABC transporters; Bile secretion

Peak area intensity were presented as mean ± standard deviation; RT, retention time; *m*/*z*, mass to charge ration; VIP, variable importance in partial least squares project; PC, Phosphatidylcholine; LysoPE, Lysophosphatidylethanolamine. Mean values were significantly different from those of the dextrose control group: *p < 0.05, **p < 0.01. Mean values were significantly different from those of the alcohol control group: ^#^ *p* < 0.05, ^##^ *p* < 0.01.

## Supplementary Materials

Supplementary materials can be accessed at: http://www.mdpi.com/1420-3049/21/4/435/s1.

## Abbreviations

The following abbreviations are used in this manuscript:

ALB	Albumin;
ALT	Alanine aminotransferase;
AST	Aspartate aminotransferase;
BCAA	Branched-chain amino acids;
ESI	Electrospray ionization source;
GC	Gas chromatography;
GLB	Globulin;
GSH	Reduced glutathione;
GSSG	Oxidized glutathione;
LCAT	Lecithin cholesterol acyltransferase;
LysoPE	Lysophosphatidylethanolamine;
MDA	Malondialdehyde;
MS	Magnetic resonance;
*m*/*z*	Mass to charge ration;
NTs	Nucleotides;
PC	Phosphatidylcholine;
PCA	Principal component analysis;
PLS-DA	Partial least squares discriminant analysis;
ROS	Reactive oxygen species;
RT	Retention time;
SFA	Saturated fatty acid;
SOD	Superoxide dismutase;
TBIL	Total bilirubin;
TC	Total cholesterol;
TG	Triglyceride;
TP	Total proteins;
UPLC	Ultra-performance liquid chromatography;
UPLC-Q-TOF-MS	Ultra-performance liquid chromatography quadrupole-time-flight mass spectrometry;
VIP	Variable importance in partial least squares project.
