# Supplementary material for: Dietary Nucleotides Supplementation and Liver Injury in Alcohol-Treated Rats: A Metabolomics Investigation
Source: Molecules. 2016 Mar 31;21(4):435. doi: 10.3390/molecules21040435 (PMC6273469; doi:10.3390/molecules21040435)
Supplement: Supplementary file 1 [file molecules-21-00435-s001.pdf]

Supplementary Materials: Dietary Nucleotides Supplementation and Liver Injury in Alcohol-Treated Rats: A Metabolomics Investigation

Xiaxia Cai, Lei Bao, Nan Wang, Meihong Xu, Ruixue Mao and Yong Li

Table S1. Waters Acquity UPLC gradient elution program applied for UPLC-Q-TOF-MS analysis.

Time (min)	Mobile Phase A (vol%)	Mobile Phase B (vol%)
Initial	100	0
1	100	0
5	40	60
8	0	100
14	0	100
16	100	0

vol, volume.

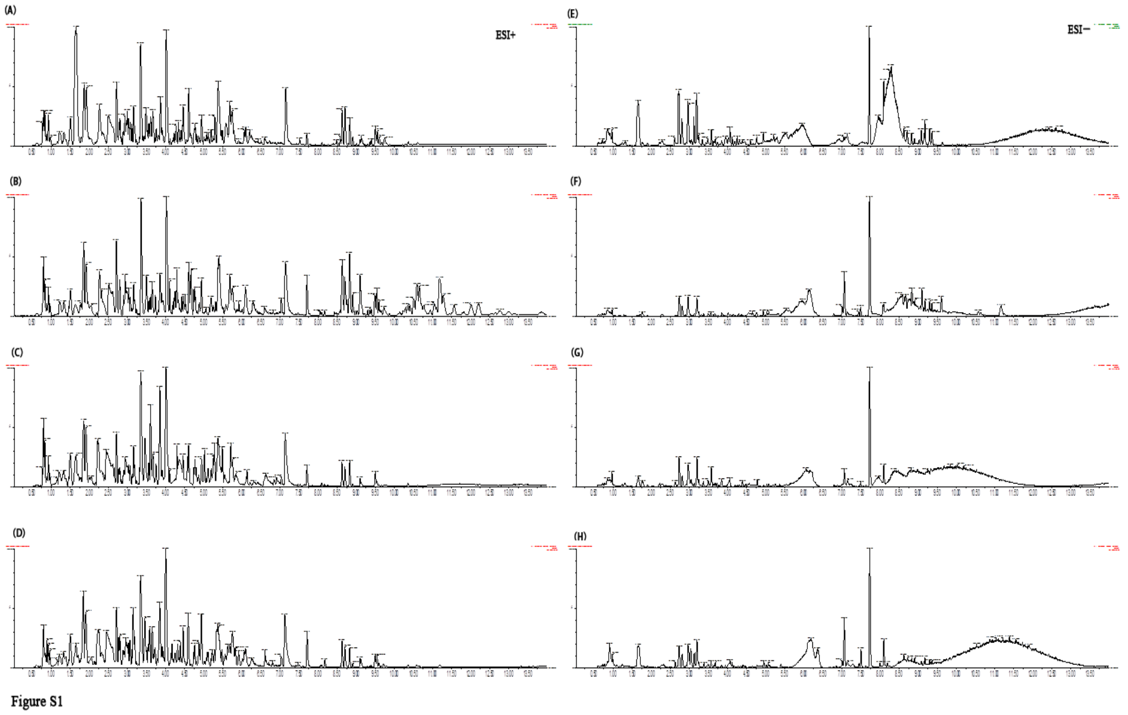

Figure S1. Base peak intensity (BPI) chromatograms obtained from the liver samples of rats. BPI chromatograms from (**A**) dextrose control group, (**B**) alcohol control group; (**C**) 0.04%NTs group and (**D**) 0.16%NTs group with positive ion mode; BPI chromatograms from (**E**) dextrose control group; (**F**) alcohol control group; (**G**) 0.04%NTs group and (**H**) 0.16%NTs group with negative ion mode.
